# Evaluating the renoprotective effects of omega-3-6-9 against cisplatin-induced nephrotoxicity in mice

**DOI:** 10.25122/jml-2023-0078

**Published:** 2023-12

**Authors:** Saja Kareem Abd Alhusen, Ali Faris Hasan

**Affiliations:** 1 Department of Pharmacology and Toxicology, College of Pharmacy, University of Baghdad, Baghdad, Iraq

**Keywords:** cisplatin, anti-inflammatory agents, cytokines

## Abstract

Fatty acids, particularly omega-3, omega-6, and omega-9, play a vital role in various biological processes. As the body cannot synthesize omega-3 and omega-6, dietary sources of these fatty acids are essential. Each omega fatty acid has a distinct chemical structure, source, and function. Cisplatin (CP) treatment is known to cause acute kidney injury (AKI) due to its inflammatory effects. This study explored the renoprotective potential of omega-3-6-9 when co-administered with cisplatin in a mice model. We divided adult mice into five groups: a control group received 0.5 ml of liquid paraffin; a cisplatin-only group; two groups were treated with low (50 mg/kg) and high (100 mg/kg) doses of omega-3-6-9 plus cisplatin; and a final group received vitamin E before cisplatin administration. The administration of omega-3-6-9 significantly decreased pro-inflammatory modulators and kidney function markers such as TNF-α, IL-1β, blood urea nitrogen, and creatinine, indicating potential renoprotective effects. Our research concluded that omega-3- 6- 9 had anti-inflammatory properties and was effective against the harmful effects of cisplatin.

## INTRODUCTION

Cisplatin-based anti-cancer treatments are important for treating various forms of cancers, such as ovarian, stomach, breast, bladder, and lung cancers [1, 2]. However, one of the adverse effects of cisplatin treatment is nephrotoxicity, which can be serious enough to warrant discontinuation of chemotherapy treatment [3, 4]. Nephrotoxicity, characterized by kidney damage, is a common complication observed across various animal models, limiting the clinical usefulness of this medicine. The kidneys play a significant role in the excretion of cisplatin. The odds of nephrotoxicity are higher following cisplatin administration due to the deposition and availability of cisplatin within proximal tubular epithelial cells being roughly five times that of the serum concentrations [5]. Therefore, identifying alternative treatments to prevent nephrotoxicity is important for patients undergoing cisplatin therapy. Studies have shown that the liver and other body organs, except the kidney, tend to accumulate cisplatin at lower levels than the kidneys do [6]. The excessive retention of cisplatin in the kidney's tissues causes cisplatin-induced nephrotoxicity.

Alpha-tocopherol, one of the eight isoforms of vitamin E, is the most powerful naturally occurring fat-soluble antioxidant. This isoform has been associated with a range of therapeutic effects, including anti-cancer properties, neuroprotective effects against Alzheimer's disease, and other protective effects [7, 8]. Alpha-tocopherol has effectively reduced oxidative stress parameters induced by L-thyroxine-induced stress [9]. Triple-omega 3-6-9 is a combination of unsaturated fatty acids. These essential fatty acids are integral components of cell membrane phospholipids, with the highest concentration in the central nervous system. They play a critical physiological role in maintaining bodily homeostasis and ensuring proper cephalogenesis [10]. Omega-3-6-9 is an alternative treatment with protective properties in preventing cisplatin-induced nephrotoxicity in mice. However, additional research is necessary to fully understand its safety and efficacy as a treatment for nephrotoxicity caused by cisplatin.

Omega-3 fatty acids, such as docosahexaenoic acid, eicosapentaenoic acid, and long-chain alpha-linolenic acid (DHA), cannot be produced by the body and must be obtained from diet. As a result, they are considered essential fatty acids. Long-chain omega-6 fatty acids, such as linoleic, gamma-linolenic, and arachidonic acids, are other examples of necessary fatty acids that can potentially be medicinal when used in moderation [11, 12]. Several researchers believe a delicate balance of fatty acid consumption is necessary because an unbalanced n-3 to n-6 ratio or too many n-6 fatty acids may promote cancer, thrombus formation, autoimmune and inflammatory diseases, cardiovascular disease, and other diseases [13, 14]. Concerning omega-9 fatty acids, oleic acid is one of them, and these fatty acids make up more than 80% of the oil in olives. Additionally, oleic acid affects the biochemical and functional attributes of cellular membranes in numerous tissues and is among the most abundant neuron membrane phospholipids [15].

Cisplatin therapy is associated with the activation of inflammatory pathways, such as the phosphorylation of nuclear factor kappa-B (NF-κB), toll-like receptors, and poly ADP-ribose polymerase-1 (PARP-1) [16]. Currently, magnesium supplements and hydration regimes are used to treat cisplatin-induced kidney damage (because cisplatin results in hypomagnesemia) [17]. Additionally, patients receiving high doses of cisplatin are treated with mannitol-induced forced diuresis. However, mannitol therapy causes over-diuresis, raising dehydration risk [17]. Therefore, finding a new medication that offers protection and is safe and effective for patients to use with cisplatin therapy is important. The objective of the current investigation was to assess any potential anti-inflammatory effects of omega-3-6-9 in mice compared to cisplatin.

## MATERIAL AND METHODS

### Animals

Thirty-five mice, with an average weight of 30 grams, were included in this study. These mice were housed in the animal facility of our college, where they were maintained under standard conditions of temperature, humidity, and light-dark cycles and provided with ad libitum access to water and a consistent diet.

### Chemicals and drugs

Omega-369 was purchased from Source Adrien Gagnon, liquid paraffin from Riedel-de Haan, and normal saline 0.9% from Pioneer. Cisplatin (1 mg/mL, 50 mL vial) was obtained from Accord. TNF and IL-1β ELISA kits were acquired from Nanjing Pars Biochem. Alpha-tocopherol was derived from natural sources in accordance with the United States Pharmacopeia (USP) standards.

### Experimental design

The mice were divided into five groups, each consisting of seven animals. Each group was assigned specific treatments as follows:

1. **Group 1 - Negative Control (NC):** Mice in this group received a daily oral administration of 0.5 ml of liquid paraffin for seven consecutive days.
2. **Group 2 - Positive Control Group (PC):** Animals in this group were subjected to a single intraperitoneal injection of cisplatin (10 mg/kg IP) as part of the experimental procedure [18].
3. **Group 3 - Omega-369 Treatment (O-369):** On the eighth day, after seven days of receiving oral omega-369 (50 mg/kg), mice in this group were administered a single intraperitoneal injection of cisplatin (10 mg/kg).
4. **Group 4 - High-Dose Omega-369 Treatment (HD O-369):** Following seven days of continuous oral intake of omega-369 at 100 mg/kg, mice in this group received a solitary intraperitoneal injection of cisplatin (10 mg/kg) on the eighth day.
5. **Group 5 - Alpha-Tocopherol Treatment Vitamin E (AT):** After seven consecutive days of oral alpha-tocopherol administration (100 mg/kg), mice in this group received a solitary intraperitoneal dose of cisplatin (10 mg/kg) on the eighth day [19-24].

After a twenty-four-hour interval since the last administered dose, blood samples were collected from the mice, which were subsequently euthanized by cervical dislocation. Both kidneys were then isolated for homogenization.

### Preparation of tissue homogenate sample

**Figure 1:**
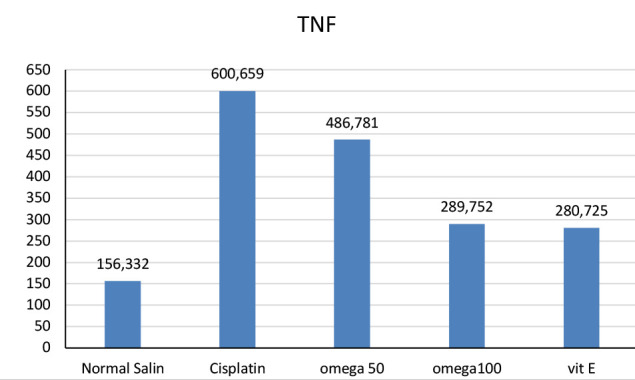
TNF-α levels across groups

**Figure 2:**
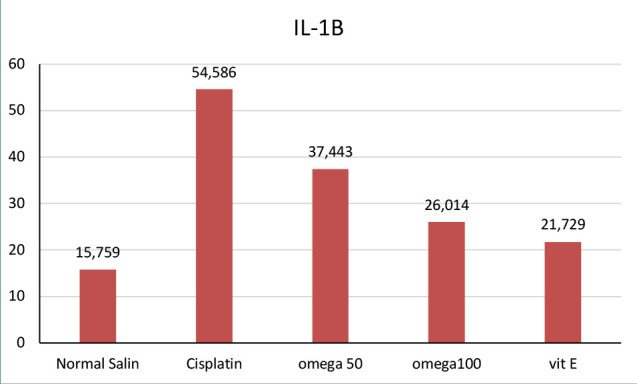
IL-1β levels across groups

Both kidneys were removed, rinsed with normal cold saline, and weighed with an electrical homogenizer before being placed in a plain tube containing 2.7 ml of phosphate-buffered saline (PBS). The homogenate was then centrifuged with a cold centrifuge for 20 minutes at 14,000 rpm, and the fluid was collected and frozen for later use to measure TNF-α and IL-1β (Figures 1, 2).

### Blood sampling and biochemical analysis

**Figure 3:**
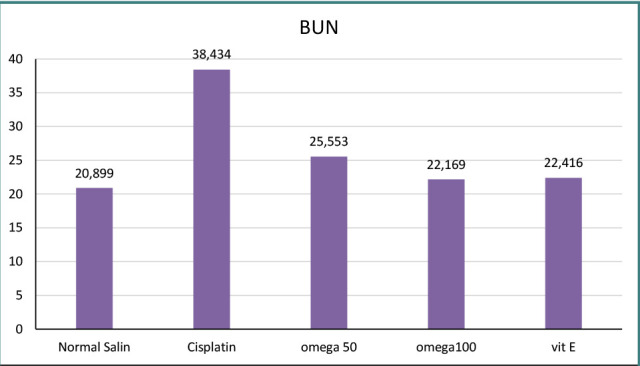
BUN levels across groups

Blood samples were collected from the jugular vein using a 25 G needle, and care was taken to extract the samples slowly to prevent the collapse of blood vessels. Subsequently, the blood was transferred into gel tubes and allowed to stand for 30 minutes to allow clot formation. After clotting, the tubes were centrifugated for 30 minutes. This allowed the serum to be separated and refined. The collected serum was stored at a temperature of -20 °C until the day of analysis. The stored serum samples were utilized to measure the levels of blood urea nitrogen (BUN) and creatinine (Figure 3).

### Statistical analysis

The data analysis was performed using the Statistical Package for Social Sciences (SPSS), version 25. Descriptive statistics were calculated for the study variables, including means and standard deviations (SD). Group differences were evaluated using an independent t-test. A significance level of 0.05 was considered statistically significant.

## RESULTS

### Effect of omega 3-6-9 on TNF-α and IL-1β among groups

The impact of omega-369 administration on TNF-α and IL-1β, two inflammation modulators, was evaluated (Figures 1 and 2). TNF-α and IL-1β levels significantly increased following cisplatin administration, indicating the ability of the drug to induce inflammation. However, treatment with omega-3-6-9 significantly decreased the levels of these inflammatory markers. The 100 mg/kg dosage of omega-3-6-9 had a more substantial reduction than 50 mg/kg. Additionally, alpha-tocopherol, administered at 100 mg/kg, demonstrated efficacy, approaching the effectiveness of omega-369 at the exact dosage.

### Effect of omega-3-6-9 on kidney function

**Figure 4:**
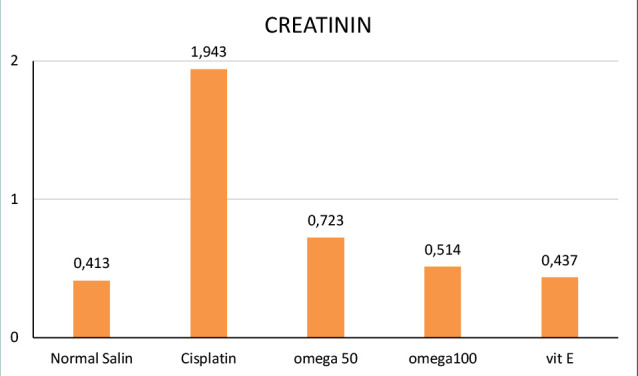
Creatinine levels across groups

The assessment of renal biochemical parameters at different intervals (Figures 3 and 4) revealed a significant increase in creatinine and BUN levels following cisplatin administration, indicating renal dysfunction. However, after the omega 3-6-9 therapy, creatinine and BUN levels were significantly reduced (Figure 4).

## DISCUSSION

Cisplatin has emerged as a panacea for treating all types of cancers [25]. However, exceeding the recommended dosage may lead to infrequent yet severe side effects such as hepatotoxicity [26]. Its use is associated with certain limitations, such as the potential for nausea, vomiting, electrolyte imbalances, hemolytic anemia, and neurotoxicity [27]. Nephrotoxicity is a grave concern, being the primary dose-limiting adverse effect of cisplatin [28]. The application of cisplatin therapy has been linked to the onset of nephrotoxicity, as evidenced by raised serum BUN and creatinine levels, indicating renal impairment. Recent research has shown that this is partly due to the stimulation of inducible nitric oxide synthase (iNOS) production. Cisplatin nephrotoxicity is partly caused by an initial inflammatory reaction. Renal disorders, such as nephropathy and other related types of kidney disease and renal failure, commonly trigger inflammation. Cytokines, chemotactic factors, and adhesion molecules are all secreted more frequently as a response to vascular injury. In this study, the inflammatory effects of cisplatin were assessed, showing a significant increase in the levels of TNF-α and IL-1β. Previous research indicates that vascular inflammation is significantly influenced by the NF-κB-IL-6 signaling pathway [29]. Furthermore, cisplatin-induced renal injury has been consistently linked with an elevated production of TNF-α in the kidneys [30-33]. The pro-inflammatory cytokine TNF-α is integral to the inflammation that cisplatin exacerbates. It facilitates the activation of other inflammatory cytokines, such as IL-1, and increases the mobility of inflammatory cells in cisplatin-induced kidney damage [34, 35].

The study found that cisplatin injection significantly increased TNF-α and IL-1β activity, causing acute inflammation compared to the negative control. These findings are consistent with previous studies that reported elevated TNF-α and IL-1β following cisplatin therapy [36-39]. Our study found that a high dose of omega-3-6-9 (100 mg/kg) significantly reduced TNF-α, IL-1β, BUN, and creatinine levels. Conversely, a lower dose of 50 mg/kg was less effective in achieving these outcomes. Recent research suggests that diets high in monounsaturated fatty acids may have anti-inflammatory effects on inflammatory bowel disease. This is supported by several studies from Iraq and other regions, which indicate that specific lipid compositions can exert a functional impact on intestinal inflammation [40-43]. Omega-3-6-9 demonstrates its anti-inflammatory effects by reducing the activity of TNF-α and IL-1β, two key inflammatory markers. Additionally, it helps lower elevated levels of BUN and creatinine induced by cisplatin, ultimately contributing to improved renal function.

## CONCLUSION

The study highlights the significant role of inflammation in cisplatin-induced nephrotoxicity and the potential of omega-3-6-9 to mitigate the adverse effects of cisplatin therapy. The study showed that omega-3-6-9 had an anti-inflammatory effect, reducing the levels of pro-inflammatory cytokines TNF-α and IL-1β and improving renal function by lowering BUN and creatinine levels. These findings suggest that omega-3-6-9 may be a promising adjuvant therapy for patients undergoing cisplatin treatment, particularly those at risk of developing nephrotoxicity. Further research is needed to validate these findings and optimize the dosages of omega-3-6-9 for effective clinical use.
